# Dissociable dorsal medial prefrontal cortex ensembles are necessary for cocaine seeking and fear conditioning in mice

**DOI:** 10.1038/s41398-024-03068-7

**Published:** 2024-09-23

**Authors:** Shuai Liu, Natalie Nawarawong, Xiaojie Liu, Qing-song Liu, Christopher M. Olsen

**Affiliations:** 1 Department of Pharmacology & Toxicology, Neuroscience Research Center, Medical College of Wisconsin, Milwaukee, WI USA; 2 Departments of Pharmacology & Toxicology and Neurosurgery, Neuroscience Research Center, Medical College of Wisconsin, Milwaukee, WI USA; 3 Department of Neurobiology, University of Maryland School of Medicine, Baltimore, MD USA; 4 Department of Pharmacology & Toxicology, The University of Texas at Austin, Austin, TX USA

**Keywords:** Neuroscience, Physiology

## Abstract

The dorsal medial prefrontal cortex (dmPFC) plays a dual role in modulating drug seeking and fear-related behaviors. Learned associations between cues and drug seeking are encoded by a specific ensemble of neurons. This study explored the stability of a dmPFC cocaine seeking ensemble over 2 weeks and its influence on persistent cocaine seeking and fear memory retrieval. In the first series of experiments, we trained TetTag *c-fos*-driven-EGFP mice in cocaine self-administration and tagged strongly activated neurons with EGFP during the initial day 7 cocaine seeking session. Subsequently, a follow-up seeking test was conducted 2 weeks later to examine ensemble reactivation between two seeking sessions via c-Fos immunostaining. In the second series of experiments, we co-injected viruses expressing TRE-cre and a cre-dependent inhibitory PSAM-GlyR into the dmPFC of male and female *c-fos*-tTA mice to enable “tagging” of cocaine seeking ensemble or cued fear ensemble neurons with inhibitory chemogenetic receptors. These c-*fos*-tTA mice have the *c-fos* promoter that drives expression of the tetracycline transactivator (tTA). The tTA can bind to the tetracycline response element (TRE) site on the viral construct, resulting in the expression of cre-recombinase, which enables the expression of cre-dependent inhibitory chemogenetic receptors and fluorescent reporters. Then we investigated ensemble contribution to subsequent cocaine seeking and fear recall during inhibition of the tagged ensemble by administering uPSEM792s (0.3 mg/kg), a selective ligand for PSAM-GlyR. In both sexes, there was a positive association between the persistence of cocaine seeking and the proportion of reactivated EGFP+ neurons within the dmPFC. More importantly, inhibition of the cocaine seeking ensemble suppressed cocaine seeking, but not recall of fear memory, while inhibition of the fear ensemble reduced conditioned freezing but not cocaine seeking. The results demonstrate that cocaine and fear recall ensembles in the dmPFC are stable, but largely exclusive from one another.

## Introduction

The prefrontal cortex is a brain region that promotes behavioral responses based on prior learned associations [1–3]. Disease states such as substance use disorder (SUD) and post-traumatic stress disorder (PTSD) involve maladaptive responses based on learned associations between a stimulus (e.g., sight of drug paraphernalia, sound of a gunshot) and action (e.g., procure drug, take cover). The link between these prior learned associations and future behavioral responses is thought to exist in the prefrontal cortex, specifically as memories stored within neuronal ensembles [4–7]. Neuronal ensembles are postulated to function as memory engrams, each representing a unique learned association between environmental cues and behavior [8–10]. Future therapeutic strategies may be able to target specific ensembles, but the effects of ensemble manipulation on other tasks, behaviors, or functions subserved by the targeted brain region are unknown.

The dorsal medial prefrontal cortex (dmPFC) is critical for promoting cocaine seeking and fear-related behaviors [11–16]. Interventions such as the infusion of pharmacological inactivators or dopamine receptor antagonists into the dmPFC result in decreased cocaine seeking behavior [17, 18] as well as a reduction in conditioned fear expression [19–21]. Despite these similar effects of dmPFC inhibition on cocaine seeking and recall of conditioned fear memories, there is evidence that distinct projection neurons from the dmPFC mediate the two behaviors. While cocaine seeking is primarily driven by the projection to the nucleus accumbens (NAc), fear recall is dependent on the projection to the amygdala [22–25]. However, it is unknown if there is sufficient dissociation of the neural encoding in the dmPFC that manipulations in this region to disrupt one behavior could leave the other unaffected.

Activity-dependent reporters using immediate-early gene promotors such as *c-fos* have been used to identify and manipulate ensemble neurons [8, 26, 27]. Ablation of a dmPFC c-Fos-expressing cocaine seeking ensemble significantly attenuated active responses in a subsequent cocaine seeking test [28]. Similarly, optogenetic inhibition of fear recall induced dmPFC ensemble neurons substantially impaired subsequent fear memory retrieval [29]. In this study, we targeted neuronal ensembles within the dmPFC to define the functional overlap between neurons encoding cocaine seeking and fear recall. We hypothesized that specificity of cocaine seeking and fear recall ensembles would be demonstrated by selectivity of ensemble inhibition to suppress the same behavioral response, but not others. We further hypothesized that this selectivity would be observed as high overlap when the same behavior was tagged on two occasions (i.e. cocaine seeking and cocaine seeking), but minimal overlap when different behaviors were tagged (i.e. cocaine seeking and fear recall). To achieve this, we used two strategies. The first strategy used TetTag mice, which are generated by breeding c-*fos*-tTA and TRE-H2B-EGFP mice together. In this Tet-off ensemble tagging system, feeding doxycycline (Dox) diet inhibits the binding of the *c-fos* promoter-driven tTA (tetracycline transactivator) to the TRE (tet-responsive element) site, thus preventing ensemble tagging. When Dox is removed from the diet, the c-fos driven rTA binds the TRE and drives expression of the histone H2B-EGFP fusion protein, thus tagging ensemble cells. Fos expression was used to identify ensembles activated during a subsequent, allowing for measurement of ensemble reactivation (EGFP + /Fos+ cells). In the second strategy, the c-*fos*-tTA mice were injected with adeno-associated viruses (AAVs) encoding TRE-cre and the cre-dependent inhibitory chemogenetic receptor PSAM-GlyR-EGFP [30] into the dmPFC. This strategy tagged neuronal ensembles activated during the initial behavioral phase with inhibitory chemogenetic receptors and an EGFP reporter. In the absence of Dox, activation of the *c-fos* promoter triggers the expression of tTA, which binds to TRE, driving the expression of cre-recombinase. The cre-dependent transgene enables the activated specific neuronal ensembles to express both inhibitory the chemogenetic receptor PSAM-GlyR and an EGFP reporter. This inhibitory PSAM-GlyR chemogenetic receptor is a chimeric protein that fuses the modified ligand-binding domain of the α7 nicotinic receptor with the pore domain of the glycine receptor. Advantages of the PSAM-GlyR system include the use of a highly selective and potent ligand (uPSEM792s: in vivo dose is <1 mg/kg), and hyperpolarization of neurons without reliance on other signaling molecules [30]. Thus, administration of uPSEM792s inhibits the activity of tagged ensemble neurons, which express the PSAM-GlyR.

## Methods

A more detailed description of methods is provided in the Supplementary Methods.

### Subjects

Male and female TetTag and *c-fos*-tTA mice were generated in house for use and began experiments at 8–20 weeks of age. Sample sizes were chosen based on our preliminary studies. Six TetTag and 22 *c-fos*-tTA mice were removed due to loss of catheter patency, leaving *n* = 14 (6–8/sex) TetTag mice and *n* = 66 (8–9/group) *c-fos*-tTA mice in the studies. Mice were handled and maintained on a reverse light cycle as described [31–34]. Mice were born and raised on doxycycline (dox) chow (40 mg/kg, Teklad Custom Research Diets, cat#120240, Envigo, Harlan Laboratories, Madison, WI) to prevent EGFP expression. After weaning, Mice were maintained, and group housed on a 12-h reverse light cycle with food and water available *ad libitum*. All animal procedures were performed in accordance with the Medical College of Wisconsin animal care committee’s regulations and the NIH Guide for the Care and Use of Laboratory Animals (8th edition). All experimental protocols were approved by the Medical College of Wisconsin Institutional Animal Care and Use Committee (protocol #AUA2698).

### Viral constructs and stereotaxic surgery

To label the cocaine seeking ensemble in the dmPFC of *c-fos*-tTA mice, a co-injection of AAV_5_-hsyn-FLEX-PSAM-GlyR-IRES-EGFP (AAV-FLEX-PSAM-GlyR-EGFP; Addgene, #119741) and AAV1-TRE-Cre (SignaGen Laboratories, #SL101511) was conducted before jugular surgery. Injections into the dmPFC were 400 nl in volume, infused 60 nl/min into the following coordinates: AP 1.7 mm; ML ± 0.4 mm; DV −2.3 mm.

### Jugular catheterization surgery

Approximately 7 days after stereotaxic surgery, mice were implanted with a silicone catheter into the right jugular vein, which exited through the intrascapular region and was connected to a cannula assembly (as described in [35, 36]). Mice were allowed to recover ≥7 days prior to the start of self-administration experiments.

### Drugs

Cocaine HCl (NIDA Drug supply) was dissolved in 0.9% saline. A stock solution of cocaine at 2 mg/ml was prepared for jugular infusions (0.5 mg/kg/infusion). uPSEM792s HCl (Hello Bio, #HB8542) was dissolved in 0.9% sterile saline and prepared on the day of use at a dosage of 0.3 mg/kg via intraperitoneal injection. Investigators were blind to ligand treatment.

### Cocaine self-administration (SA)

Cocaine SA was performed as described [35] with minor modifications. After the mice had fully recovered from jugular catheterization surgery, they acquired cocaine SA on a fixed ratio-1 (FR-1) schedule for 7–14 days. During the SA session, a single press on the active lever resulted in the delivery of cocaine (0.5 mg/kg/infusion) and cue light illumination, followed by a 10 s timeout. Presses on the active lever during timeout or on the inactive lever at any time were recorded but had no programmed consequence. SA sessions were 3 h in duration but ended early if the limit of 64 infusions was achieved prior to the end of the session. Cocaine self-administration training continued until specific criteria were met (four consecutive sessions of >20 reinforcers and a 2:1 ratio of active to inactive lever presses, minimum of seven sessions). If criteria were not met following the initial seven sessions, catheter patency was assessed using Brevital (methohexital, 9 mg/kg, iv) and any mouse not meeting criteria for patency (sedation within 5 s) was excluded from the study. Self-administration sessions were conducted for a maximum of 14 days.

### Cocaine seeking

On the 7th day of forced abstinence, mice were placed back in the operant chambers and underwent a 2 h cocaine seeking session under extinction conditions, during which the ensemble was tagged (see supplementary methods). This seeking session was under the same conditions as cocaine SA (pump and cue light), except no cocaine infusions were delivered and sessions were 2 h in duration, regardless of the number of lever presses. On the 21st day of abstinence, all mice underwent a second cocaine seeking session. This session was identical to the one performed on day 7. For *c-fos*-tTA mice, the uPSEM792s ligand (0.3 mg/kg) or vehicle (saline) was administered 30 min prior to the start of the second seeking session.

### Slice preparation and electrophysiology

Twenty-four hours after the day 21 seeking session, two of the *c-fos*-tTA mice were sacrificed and brain slices containing the dmPFC were prepared. Whole-cell recordings were conducted from dmPFC layer V/VI pyramidal neurons that were either EGFP+ or EGFP-. A 20-pA current injection was applied to induce stable action potential firing in the neurons. Pressure injection of the uPSEM792s ligand (50 nM) was given via a glass pipette (1–2 µm tip opening, 5 psi, 2 s) in close proximity to the recorded neuron. More details can be found in the Supplementary Methods.

### Novel open field test

Mice were placed into the center of a circular open field chamber. Total distance traveled, entries into center and immobile time were calculated through an automated video-tracking system (ANY-maze, Stoelting, Wood Dale, IL). Thirty minutes prior to the assay, the mice received either an uPSEM792s ligand (0.3 mg/kg) or a vehicle.

### Fear conditioning

Mice underwent a single fear conditioning training session similar to other reports [37–39]. The session consisted of seven pairings of tones and foot shocks. The following day, they were evaluated in a cued memory retrieval test conducted in a novel context. During this test, mice were exposed to seven tones without foot shocks. Consistent with previous sessions, uPSEM792s ligand (0.3 mg/kg) or vehicle was administered to the animals 30 min prior to the test.

### Immunohistochemistry and image analysis

Immediately following the second cocaine seeking session or 1-h after the end of the cued fear test, mice were subjected to transcardial perfusion. After fixation, coronal sections (20 μm) were obtained using a cryostat for subsequent c-Fos immunostaining. dmPFC sections in all the experiments were imaged using a Leica SP8 confocal microscope and images were analyzed automatically using Imaris 10.0 (Bitplane/Oxford Instruments, Abington, England) software to count the EGFP+ and c-Fos+ nuclei with the “spots” function.

### Statistical analysis

Prism10 (GraphPad, San Diego, CA, USA) or SPSS 28 (IBM, Armonk, NY) were used for statistical analyses. Data were analyzed by Student’s *t*-test, linear regression, ANOVA (repeated measures when appropriate), linear mixed effect analysis, or ANCOVA followed by Holm-Sidak multiple comparisons tests. A value of *p* ≤ 0.05 was considered significant.

## Results

### Relationship between mPFC cocaine seeking ensemble reactivation and persistence of cocaine seeking behaviors

**Fig. 1:**
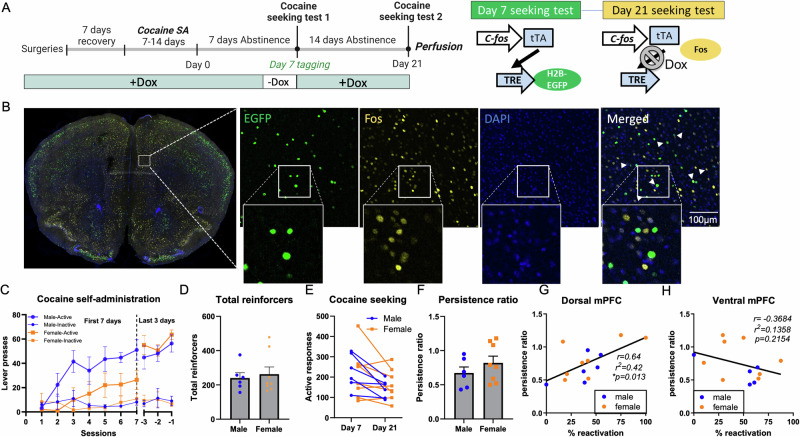
Reactivation of neural ensembles in the dmPFC during cocaine seeking sessions. **A** Experimental timeline and procedures to tag cells activated by cocaine seeking in the dmPFC of TetTag mice. Mice performed cocaine self-administration sessions, then two drug seeking tests. Three days prior to the first seeking test, all mice were removed from Dox diet and placed on standard lab chow until after the early drug seeking session to open the tagging window. All mice were sacrificed immediately after the second cocaine seeking test for c-Fos immunostaining. Right: Dox-regulated tetracycline-transactivator (tTA) system for ensemble labeling. Mice carrying two transgenes were used. The first transgene uses the *c-fos* promotor to drive expression of tTA. tTA activates the TetO response element (TRE) in the absence of Dox. The second transgene uses the TRE promoter to drive expression of a histone H2B-EGFP fusion protein. During the second seeking test, the *c-Fos* promoter drives expression of tTA, however, when Dox is in present, tTA is unable to bind to TRE and drive expression of H2B-EGFP. **B** Representative images of the first seeking test ensemble (EGFP+ cells) and the second seeking test ensemble (c-Fos+ cells) in the dmPFC. EGFP+ cells comprised 1.9 ± 0.34% and c-Fos+ cells comprised 7.2 ± 0.78% of DAPI cells. **C** Lever presses by male (blue, *n* = 7) and female (orange, *n* = 8) TetTag mice during cocaine self-administration. The first 7 days refer to the average active and inactive lever presses during the initial 7 days of SA. The last 3 days refer to the average active and inactive lever presses during the final 3 days of SA. **D** Total number of cocaine reinforcers earned by male and female mice. **E** Active lever presses on the first (Day 7) and second (Day 21) cocaine seeking tests. **F** Cocaine seeking persistence ratio, defined as Day 21/Day 7 active lever presses. **G** Correlation between the percentage of EGFP+ cells co-expressing c-Fos+ and cocaine seeking persistence ratio in the dmPFC. **H** Correlation between the percentage of EGFP+ cells co-expressing c-Fos+ and cocaine seeking persistence ratio in the vmPFC. *N* = 6/8 male/female. Data are presented as mean ± SEM, *p < 0.05.

We first examined the relationship between ensemble reactivation and persistence of cocaine seeking by ensemble tagging an initial seeking session, then measuring reactivation of mPFC ensembles in a subsequent cocaine seeking session. Figure 1A shows the timeline and procedures in this experiment, and a representative image of EGFP and c-Fos expression in the dmPFC is shown in Fig. 1B. During the initial 7 days, active responding increased relative to inactive responding in a similar manner in both male and female mice (session x sex interaction: F(6,144) = 1.828, *p* = 0.10; main effect of lever: F(1,24) = 10.67, *p* = 0.003). Although a mixed-effects analysis revealed a main effect of sex in lever presses in the initial 7 days (main effect of sex: F(1,24) = 9.16, *p* = 0.006; sex x lever interaction: F(1,24) = 3.42, *p* = 0.08), there were no significant sex differences as measured by lever pressing during the last 3 days (sex x lever interaction: F(1,24) = 0.60, *p* = 0.45; main effect of sex: F(1,24) = 0.51, *p* = 0.48; Fig. 1C). There was also no difference in the total number of cocaine reinforcers earned by male and female mice (t(12) = 0.41, *p* = 0.69; Fig. 1D). The analysis for seeking behaviors revealed a minor reduction in active responding during the day 21 seeking session compared to the day 7 seeking session for both male and female mice, but there was no sex effect in seeking (main effect of sex: F(1,12) = 0.005, *p* = 0.94; main effect of time: F(1,12) = 10.77, *p* = 0.007; time x sex interaction: F(1,12) = 0.55, *p* = 0.47; Fig. 1E). To control for these differences, we quantified the changes in responding between session 1 and 2 as a persistence ratio. This measurement took the number of active lever presses that occurred during the second session and divided it by the number of active responses that occurred during session one. There was no difference in the persistence ratios between male and female mice (t(12) = 1.07, *p* = 0.31; Fig. 1F), therefore analyses were combined for the two sexes. Examination of cumulative lever responses during each seeking session revealed that seeking endured through the duration of each session (Supplementary Fig. 1A), validating our choice to tag the full 2 h sessions.

The main objective of this experiment was to compare the activation of a cocaine seeking neuronal ensemble across sessions. To do this, we analyzed the colocalization between EGFP+ neurons (Day 7 tagged) and c-Fos+ neurons (Day 21 tagged), identifying these double-labeled neurons as the reactivated cocaine seeking neurons that were engaged during both first and second cocaine seeking sessions. The proportion of reactivated neurons was calculated by dividing the number of double positive (EGFP + /c-Fos + ) cells by the number of EGFP+ cells. We observed 41 ± 25% ensemble reactivation in the dmPFC and 46 ± 25% ensemble reactivation in the ventral medial PFC (vmPFC). This contrasted with the 3.8 ± 3.8% reactivation observed in dmPFC and 12.7 ± 2.5% reactivation observed in vmPFC from mice that were TetTagged in the home cage and c-Fos tagged in the Day 21 cocaine seeking session. We then performed linear regressions to determine if the percentage of reactivated cells was associated with cocaine seeking persistence. There was a positive association between persistence ratio and the proportion of reactivated EGFP+ cells in the dmPFC (*r*^*2*^ = 0.42, *p* = 0.013; Fig. 1G), but not the vmPFC (*r*^2^ = 0.14, *p* = 0.22; Fig. 1H). As greater reactivation of the dmPFC ensemble was associated with higher persistence of cocaine seeking 2 weeks later, it suggests that reactivation of the dmPFC drug seeking ensemble may be necessary for subsequent cocaine seeking.

### Validation of expression and function of cre-dependent PSAM-GlyR

**Fig. 2:**
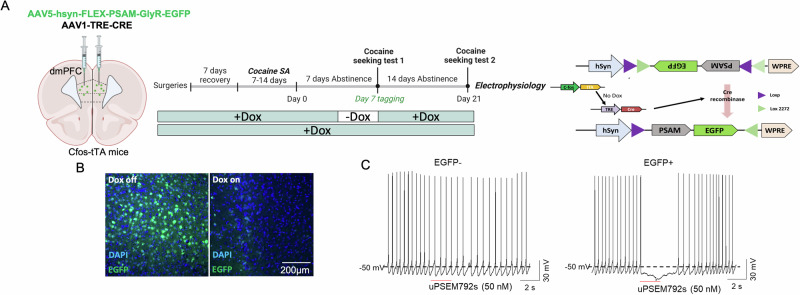
Validation of expression and function of cre-dependent PSAM-GlyR. **A** Left: Virus-based activity-dependent ensemble labeling. *c-fos*-tTA mice (male and female) were co-injected with AAV5-hsyn-FLEX-PSAM-GlyR-EGFP and AAV1-TRE-Cre viruses bilaterally targeting the dmPFC to enable “tagging” of ensemble neurons with an inhibitory PSAM-GlyR and EGFP. Middle: Experimental timeline and protocol. After stereotaxic and jugular catheterization surgeries, mice performed 3 h cocaine self-administration sessions (0.5 mg/kg) and two 2 h cocaine seeking sessions. Mice were divided into two groups (counterbalanced by cocaine intake): Tagged vs nontagged. In the tagged group, the cocaine seeking ensemble was labeled during the abstinence day 7 seeking test in the absence of Dox. In the nontagged group, Dox diet was administered for the duration of the study. Right: Mechanism of the virus-based ensemble tagging. During tagging, activation of the *c-fos* promoter results in expression of tTA and its subsequent binding to TRE, thereby driving the expression of Cre-recombinase. In the presence of Cre-recombinase, the Cre-dependent PSAM-GlyR-EGFP was expressed. **B** Representative image showing inducible activity-dependent expression of PSAM-GlyR-EGFP. Left: Image of dmPFC from a tagged animal. Right: Image of dmPFC from a non-tagged animal. **C** Validation of inhibitory PSAM-GlyR function through pressure injection of uPSEM792s ligand (50 nM). Pressure injection of uPSEM792s significantly blocked action potential firing of EGFP+ neurons expressing PSAM-GlyR, while producing no effect on EGFP- neurons without expression of these receptors.

To test whether reactivation of the dmPFC drug seeking ensemble is necessary for subsequent cocaine seeking, we used a chemogenetic ensemble tagging strategy, where *c-fos*-tTA mice were co-injected with AAV-TRE-cre and the cre-dependent inhibitory PSAM-GlyR (AAV-FLEX-PSAM-GlyR-EGFP) into the dmPFC (Fig. 2A and supplementary methods). We validated the viral strategy and PSAM-GlyR chemogenetic tool functionality by examining expression of EGFP and measuring the effects of the chemogenetic ligand on action potential firing. Mice underwent cocaine self-administration, the first cocaine seeking session was tagged, then a second seeking session was conducted as described above. A substantial number of neurons expressing EGFP was observed in the dmPFC of the tagged group, while negligible EGFP expression was detected in the dmPFC of the nontagged group that was maintained with dox during the day 7 cocaine seeking session (Fig. 2B). Additionally, we determined the function of inhibitory PSAM-GlyR on dmPFC pyramidal neurons ex vivo using whole-cell patch-clamp electrophysiology (Fig. 2C). Pressure injection of the PSAM-GlyR ligand uPSEM792s (50 nM) blocked action potential firing on EGFP+ neurons expressing with PSAM-GlyR, while producing no effect on EGFP- neurons. This confirms that the uPSEM792s selectively inhibits neuronal activity in cells expressing PSAM-GlyR.

### The dmPFC cocaine seeking ensemble is necessary for seeking memory retrieval

**Fig. 3:**
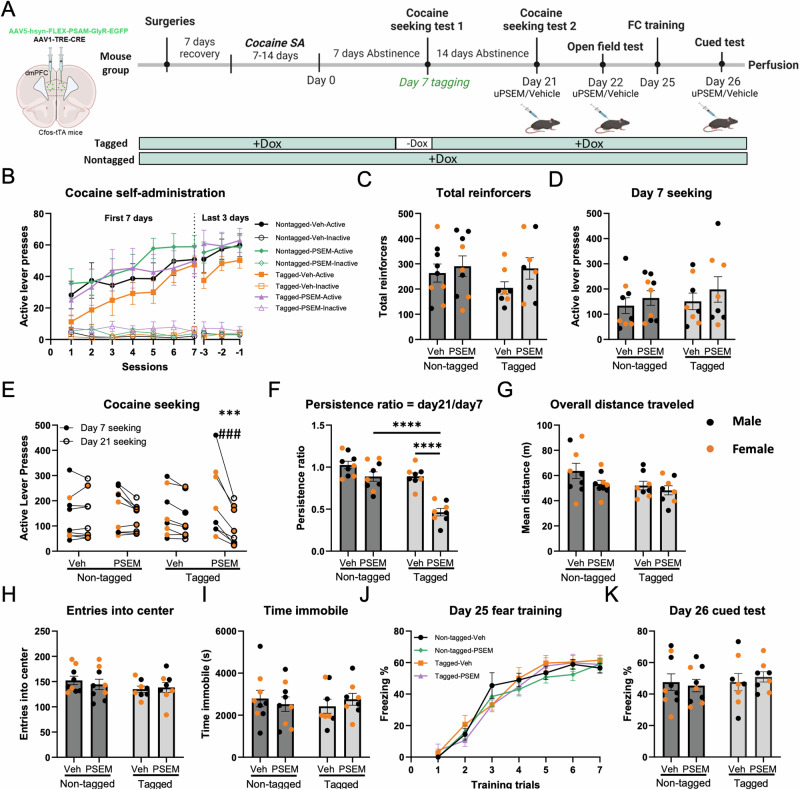
A dmPFC cocaine seeking ensemble is necessary for cocaine seeking memory retrieval, while exhibiting no discernible impact on locomotor activity and fear memory retrieval. **A** Experimental protocol for chemogenetic inhibition of dmPFC cocaine seeking ensemble. *c-fos*-tTA mice were co-injected with AAV-PSAM-GlyR-EGFP and AAV-TRE-Cre viruses into the dmPFC, then underwent cocaine self-administration (SA). Following 7 days of abstinence, mice underwent a first cocaine seeking session, then underwent a second session on day 21 of abstinence. A novel open field test was conducted 24 h after the day 21 cocaine seeking session, then locomotor activity was assessed in a novel open field test, after administration of uPSEM792s ligand or vehicle. Three days after the open field test, all mice were subjected to fear conditioning training. A cued test with seven tones under a novel context was performed 24 h after the training session with administration of ligand or vehicle 30 min prior to the test. Animals that received uPSEM792s treatment on day 21 were also administered the ligand prior to the open field test and cued test, while animals that received vehicle treatment on day 21 were similarly administered vehicle prior to the tests. Mice were sacrificed 90 min after the day 26 cued test for analysis of colocalization between ensembles encoding cocaine seeking and conditioned fear. **B** Active and inactive lever responding over the first 7 days and last 3 days of daily 3-h SA sessions. Active lever presses denoted as solid spots; inactive lever presses denoted as hollow spots. **C** Total reinforcers earned during SA sessions. **D** Active lever presses during the abstinence day 7 cocaine seeking tagging session. **E** Individual values of number of active lever presses on day 7 and day 21 seeking. Comparison of estimated marginal means found that tagged mice treated with PSEM had fewer active lever presses than the non-tagged vehicle group (***p < 0.001) and the non-tagged PSEM group (^###^*p* < 0.001). **F** Cocaine seeking persistence ratio, defined as day 21/day 7 active lever presses. **G** Distance traveled in novel open field, **H** Novel open field entries into the center zone. **I** Time immobile during novel open field. **J**, **K** The percentage of freezing time during fear conditioning training session and cued fear memory retrieval test. *N* = 18/16 male/female. Data are presented as mean ± SEM. ****p < 0.0001.

Next, we investigated the behavioral effects of inhibition of a cocaine seeking ensemble in the dmPFC. Separate male and female mice (*n* = 18/16 M/F) underwent cocaine SA followed by 7 days of abstinence (Fig. 3A timeline). All mice were assigned to one of four groups (counterbalanced by SA performance): (1) a non-tagged group (continuous dox access) with vehicle administration on the day 21 seeking session; (2) a non-tagged group with uPSEM792s administration on the day 21 seeking session; (3) a tagged group (dox removed prior to day 7 seeking) with vehicle administration on the day 21 seeking session and (4) a tagged group with uPSEM792s administration on the day 21 seeking session. There was no significant difference between groups in active or inactive lever presses during the last 3 days of self-administration (main effects of tag, ligand, and interaction all *p* > 0.60; Fig. 3B), in total reinforcers earned (main and interaction effects all *p* > 0.36; Fig. 3C), or active lever presses during the tagged cocaine seeking session (main and interaction effects all *p* > 0.29; Fig. 3D). Figure 3E depicts the active responses of each animal in all groups during the day 7 and day 21 seeking sessions. Day 21 seeking was analyzed by 2-way ANCOVA using Day 7 seeking as a covariate. There were main effects of tag (F (1,33) = 18.8, *p* < 0.001), ligand (F(1,33) = 22.2, *p* < 0.001, and an interaction of tag and ligand (F1,33) = 5.2, *p* = 0.031). Post hoc comparisons of estimated marginal means found that the tagged group that received uPSEM792s had fewer active lever presses than the non-tagged vehicle group and the non-tagged PSEM group (both *p* < 0.001). Next, we measured the persistence ratio for each subject. There was a significant main effect of tag (F(1,30) = 34.63, *p* < 0.0001), ligand (F(1,30) = 35.22, *p* < 0.0001). and a significant interaction between tag and ligand (F(1,30) = 9.24, *p* = 0.0049). Post hoc comparisons revealed that the persistence ratio was significantly lower in tagged animals given uPSEM792s compared to non-tagged mice given uPSEM792s and tagged animals given vehicle (both *p* < 0.0001; Fig. 3F). Taken together, these findings indicate that the dmPFC cocaine seeking ensemble established 7 days after abstinence is necessary for retrieval 2 weeks later.

### Inhibition of the dmPFC cocaine seeking ensemble does not affect locomotor activity or fear memory retrieval

To assess the potential impact of temporal inhibition of dmPFC cocaine ensemble activity on locomotor activity during the second cocaine seeking session, a novel open field test was conducted 24 h after the day 21 cocaine seeking session. Locomotor activity was assessed during this 2 h open field session, initiated 30 min after i.p. administration of uPSEM792s or vehicle (Fig. 3A). Animals received the same ligand treatment for the open field test as they did on the day 21 cocaine seeking test. There were no differences between any groups in overall distance traveled (main effect of tag: F(1,30) = 3.80, *p* = 0.06; main effect of ligand: F(1,30) = 2.73, *p* = 0.11), tag x ligand interaction: F(1,30) = 0.59, *p* = 0.45; Fig. 3G, entries into center (main effect of tag: F(1,30) = 1.72, *p* = 0.20; main effect of ligand: F(1,30) = 0.06, *p* = 0.53, tag x ligand interaction: F(1,30) = 0.40, *p* = 0.53; Fig. 3H), or time immobile (main effect of tag: F(1,30) = 0.04, *p* = 0.84; main effect of ligand: F(1,30) = 0.008, *p* = 0.93, tag x ligand interaction: F(1,30) = 0.76, *p* = 0.39; Fig. 3I). Considering the near significant effects of tag on distance and ligand on center entries, we also measured inactive lever responding during the seeking session as an additional measure of non-specific changes in activity. There was no effect of tag, ligand, or interaction during the day 21 seeking test (all p > 0.41), confirming that inhibition of the cocaine seeking ensemble did not reduce cocaine seeking by non-specifically reducing locomotor activity.

Our previous findings demonstrated that inhibition of the dmPFC cocaine seeking ensemble could effectively deter subsequent cocaine seeking. However, it remains unclear whether suppressing the activity of this ensemble would alter other behaviors also mediated by the same brain region. We conducted a study to investigate the effect of inhibiting the dmPFC cocaine seeking ensemble on fear memory retrieval. The dmPFC is required for the expression, consolidation, and retrieval of fear memory [1, 13, 21, 40–42]. Fear conditioning training occurred 3 days after the open field test, and a cued test was performed 24 h after the training session with administration of uPSEM792s or vehicle that was administered 30 min prior to the cued test (timeline in Fig. 3A). There was no difference in acquisition of fear conditioning between groups (all main effects and interactions (except session main effect) *p* > 0.20), and there was no significant main effect or interaction between each group in freezing time during day 26 cued fear test (tag x ligand interaction: F(1,30) = 0.36, *p* = 0.55; main effect of tag: F(1,30) = 0.37, *p* = 0.55, main effect of ligand: F(1,30) = 0.01, *p* = 0.91; Fig. 3K). These findings indicate that suppression of the dmPFC cocaine seeking ensemble did not affect cued fear memory retrieval.

### Analysis of colocalization between ensembles encoding cocaine seeking and recall of conditioned fear

**Fig. 4:**
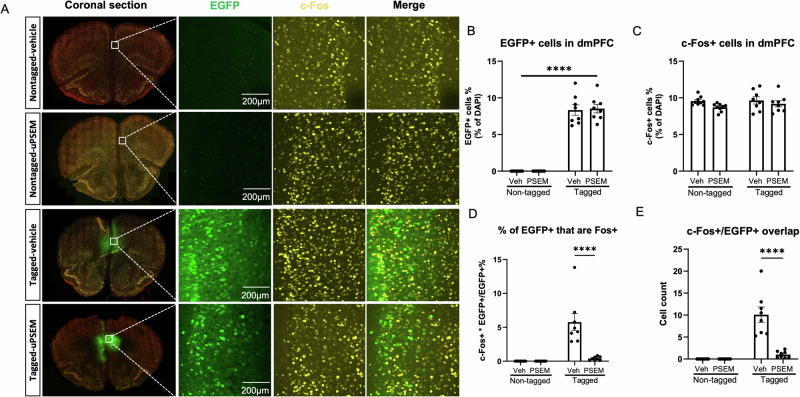
Analysis of colocalization between ensembles encoding cocaine seeking and conditioned fear in the dmPFC. **A** Example confocal images of coronal section of mPFC, EGFP+, c- Fos+, and EGFP/c-Fos double-labeled neurons in the dmPFC of *c-fos*-tTA mice from different groups. **B**, **C** Quantification of percent of EGFP+ and c-Fos+ neurons in the dmPFC. **D** Percentage of c-Fos/EGFP double-labeled EGFP+ cells of the dmPFC. **E** Cell count of cells co-expressing EGFP and c-Fos in these four groups. *N* = 8–9 mice per group. Data are presented as mean ± SEM, ****p < 0.0001.

Mice were sacrificed 90 min after the cued test for c-Fos immunostaining. Ensembles tagged during the first cocaine seeking session were EGFP+, while c-Fos+ neurons indicated the ensemble engaged in the conditioned cued fear recall test (Fig. 4A). In the tagged groups, approximately 8% of dmPFC DAPI-labeled cells were EGFP+, and there was no significant difference in the number of EGFP+ cells between these two tagged groups (*p* = 0.75; Fig. 4B), nor was there a difference in the number of c-Fos+ cells between each group (all *p* > 0.14, Fig. 4C).

Thus, inhibition of the cocaine seeking ensemble did not alter the number of c-Fos+ cells identified after the fear recall session (Fig. 4C). Of the cocaine seeking ensemble (EGFP+) cells, approximately 7% were reactivated during cued fear memory retrieval in mice that received vehicle, while ~1% were reactivated in mice that received uPSEM792s (*p* < 0.0001 for %EGFP+ that are Fos+ and cell count; Fig. 4D, E). These results indicate that <10% of the cocaine seeking ensemble cells were engaged during recall of a conditioned fear memory, and uPSEM792s was highly effective in reducing reactivation of the tagged ensemble cells. Because the overlap was so low, ensemble inhibition did not reduce the overall number of c-Fos+ cells.

### Inhibition of the dmPFC cued fear ensemble does not affect cocaine seeking

**Fig. 5:**
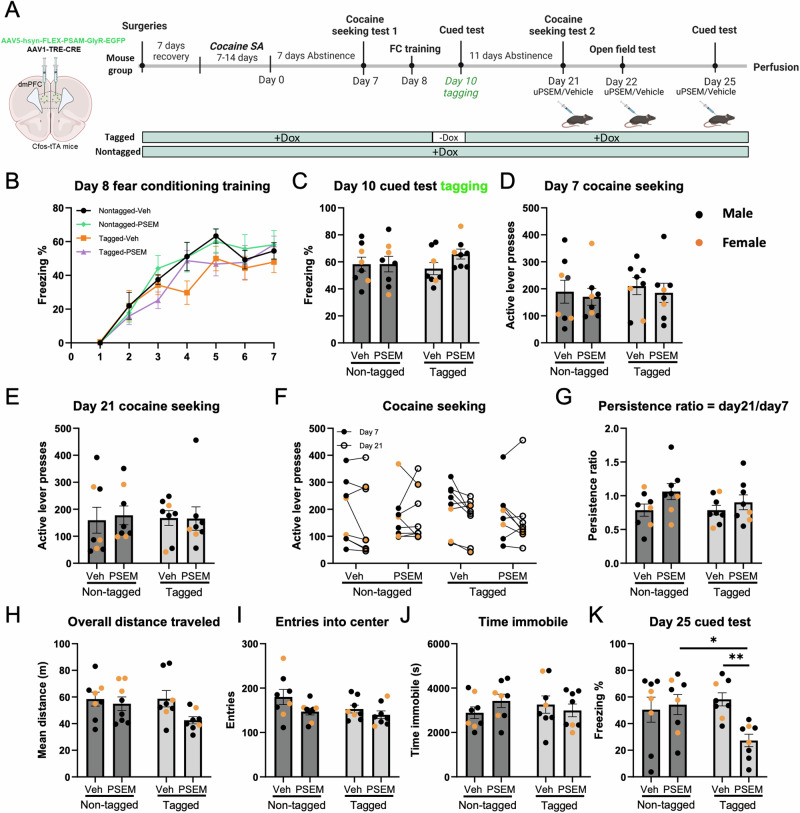
Inhibition of the dmPFC cued fear ensemble does affect cocaine seeking or locomotor activity, yet is indispensable for the subsequent retrieval of the cued fear memory. **A** Experimental protocol for chemogenetic inhibition of dmPFC cued fear ensemble. After recovery from stereotaxic and jugular catheterization surgeries, mice were subjected to cocaine SA training for a period of 7–14 days. Following the SA, all mice underwent a 7-day abstinence in their home cages. All mice were given access to Dox during day 7 seeking session since no tagging happened at this stage. Subsequently, all mice were transferred to high Dox diet to prevent tagging during the fear conditioning training session, which included foot shocks. Mice then were returned to normal Dox diet after the training session. Dox was removed 24 h before the day 10 cued test tagging phase to open the tagging window and was resumed after the cued test to stop tagging for animals in the tagged groups. Animals in the other two non-tagged groups continued to receive Dox during day 10 cued test. A second seeking session also occurred 14 days after the initial seeking session, and either vehicle or uPSEM792s ligand were given to animals in different groups 30 min prior to day 21 seeking. A novel open field test was conducted 24 h after day 21 seeking. Animals that received ligand treatment on day 21 were also administered ligand prior to the open field test, while animals that received vehicle treatment on day 21 were similarly administered vehicle prior to the test. After completing the open field test, a 30 min cued fear recall test was performed three days later with administration of either ligand or vehicle. **B** The percentage of freezing time during day 8 fear conditioning training session. **C** The percentage of freezing time during day 10 cued test tagging session (black: male; orange: female). **D**, **E** Active lever presses of individuals from each group during 2 h day 7 and day 21 cocaine seeking session. F Individual values of number of active lever presses on day 7 and day 21 seeking. **G** Cocaine seeking persistence ratio, defined as day 21/day 7 active lever presses. **H** Distance traveled in novel open field. **I** Novel open field entries into the center zone**. J** Time immobile during novel open field. **K** The percentage of freezing time during the cued fear memory retrieval test. Data are presented as mean ± SEM. *N* = 21/10 male/female. *p < 0.05, **p < 0.01.

From our results above, inhibition of a cocaine seeking ensemble in the dmPFC effectively blocked subsequent drug seeking but had no effect on novel open field locomotor activity or fear memory retrieval. To further test the hypothesis that cocaine seeking and fear memory recall are encoded by specific, dissociable dmPFC ensembles, we performed an experiment in new mice (*n* = 22/10 male/female) where a fear recall ensemble was tagged and tested the effects on ensemble inhibition on cocaine seeking, locomotor activity, and subsequent fear recall. Mice acquired cocaine self-administration, then underwent an initial drug seeking test, then fear conditioning. There were no differences between groups in cocaine SA (Supplementary Fig. 2) or fear conditioning. A three-way repeated ANOVA revealed that there was a significant main effect of session (F(6,196) = 41.22, *p* < 0.0001), while there was no significant main effect of tag or ligand or tag x ligand interaction (all *p* > 0.08) in the percentage of freezing time between groups during the fear training session (no ligand or vehicle was administered during fear conditioning; Fig. 5B). There was also no effect of group on the tagged fear recall session (main effect of tag: F(1,28) = 0.18, *p* = 0.67; main effect of ligand: F(1,28) = 1.28, *p* = 0.27; tag x ligand interaction: F(1,28) = 1.24, *p* = 0.28; Fig. 5C). After tagging the dmPFC ensemble, the effects of inhibiting the fear recall ensemble were tested on a cocaine seeking test on abstinence day 21. Inhibition of the dmPFC fear ensemble had no effect on cocaine seeking as illustrated by a lack of interaction between tagging, ligand administration, and session on active lever presses on day 21 (main effect of tag: F(1,28) = 0.002, *p* = 0.96; main effect of ligand: F(1,28) = 0.04, *p* = 0.84; tag x ligand interaction: F(1,28) = 0.07, *p* = 0.80; Fig. 5E), even when using day 7 seeking as a covariate (main effect of tag (F(1,31) = 0.78, *p* = 0.38)); main effect of ligand (F(1,31) = 1.8, *p* = 0.18); tag x ligand interaction (F(1,31) = 0.12, *p* = 0.73; Fig. 5F). Similarly, there was a lack of main effect or interaction of tagging and ligand on persistence ratio (main effect of tag: F(1,28) = 0.64, *p* = 0.43; main effect of ligand: F(1,28) = 3.87, *p* = 0.06; tag x ligand interaction: F(1,28) = 0.64, *p* = 0.43; Fig. 5G).

Ensemble inhibition also did not have an effect on novel open field (as revealed by interactions) on overall distance traveled (main effect of tag: F(1,28) = 1.50, *p* = 0.23; main effect of ligand: F(1,28) = 3.92, *p* = 0.06; tag x ligand interaction: F(1,28) = 1.64, *p* = 0.21; Fig. 5H), entries into center (main effect of tag: F(1,28) = 2.34, *p* = 0.14; main effect of ligand: F(1,28) = 4.18, *p* = 0.05; tag x ligand interaction: F(1,28) = 0.78, *p* = 0.38; Fig. 5I), or time immobile (main effect of tag: F(1,28) = 0.01, *p* = 0.92; main effect of ligand: F(1,28) = 0.18, *p* = 0.67; tag x ligand interaction: F(1,28) = 1.62, *p* = 0.21; Fig. 5J). Inhibition of the dmPFC fear recall ensemble did have an effect on subsequent fear recall, as there was a significant interaction between tagging and ligand (tag x ligand interaction: F(1,28) = 6.27, *p* = 0.02; main effect of tag: F(1,28) = 1.92, *p* = 0.18; main effect of ligand: F(1,28) = 3.84, *p* = 0.06). It should be noted that there was a main effect of ligand on novel open field center entries (*p* = 0.05) and a trend toward this main effect on distance traveled. However, non-specific reduction in locomotion would not be consistent with the decrease in freezing observed during the fear recall test.

### The dmPFC cued fear ensemble is necessary for subsequent retrieval of the cued fear memory

To determine if inhibition of the dmPFC fear ensemble would affect locomotor activity, a novel open field test was conducted 24 h after day 21 seeking (Fig. 5A). There was no significant difference between each group in overall distance traveled, entries into center, and time immobile during the test (Fig. 5H–J). Three days later, we tested the effect of inhibiting the dmPFC fear ensemble on subsequent recall of cued fear. Ensemble inhibition significantly decreased the percentage of freezing time in the tagged uPSEM792s group compared to both the tagged vehicle group (*p* = 0.003) and the non-tagged uPSEM792s group (*p* = 0.01, Fig. 5K). Taken together, these results suggest that suppression of the dmPFC conditioned cued fear ensemble inhibits subsequent cue-induced fear memory retrieval without impacting locomotor activity.

### Analysis of colocalization between ensembles activated during day 10 cued test tagging session and day 25 cued test

**Fig. 6:**
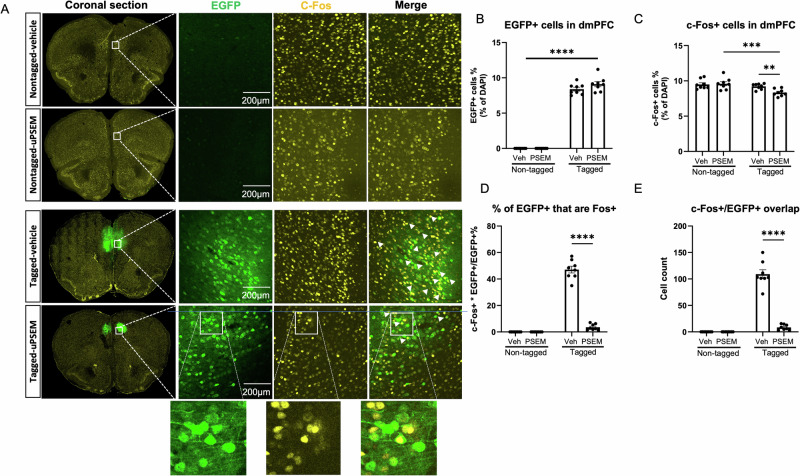
Analysis of colocalization between ensembles activated during day 10 cued fear recall test tagging session and day 25 cued fear recall test. **A** Example confocal images of coronal section of EGFP+, c-Fos+, and EGFP/c-Fos double-labeled neurons in the dmPFC of *c-fos*-tTA mice from different groups. **B**, **C** Quantification of percent of EGFP+ and c-Fos+ neurons in the dmPFC. **D** percentage of c-Fos/EGFP double-labeled cells in EGFP+ neurons in the dmPFC. Compared to the Tagged-vehicle, the percentage of c-Fos/EGFP double-labeled cells decreased significantly. **E** Cell count of cells co-expressing EGFP and c-Fos in these four groups. Compared to the Tagged-vehicle group, the cell count of c-Fos/EGFP double-labeled cells in the Tagged-PSEM groups decreased significantly (*p* < 0.0001). *N* = 8 mice/group. Data are presented as mean ± SEM, ****p < 0.0001.

Mice were sacrificed 90 min after the cued test for c-Fos immunostaining. Ensembles tagged during the first cued fear recall test were EGFP+, while c-Fos+ neurons indicated the ensemble engaged in the second conditioned cue test (Fig. 6A). In the tagged groups, approximately 8% of dmPFC DAPI-labeled cells were EGFP+ (no difference between the two tagged groups: *p* = 0.09), suggesting that the number of cells recruited to the fear recall and cocaine seeking ensembles is similar (Figs. 6B, 4B). Suppression of the tagged fear ensemble decreased the total number of c-Fos+ neurons identified in the second fear recall session (main effect of tag (F(1,14) = 13.33, *p* = 0.003)) and tag x ligand interaction (F(1,14) = 5.49, *p* = 0.03) but no main effect of ligand (F(1,14) = 2.95, *p* = 0.11; Fig. 6C, D). Of the initial fear recall ensemble (EGFP+)cells, approximately 50% were reactivated during cued fear memory retrieval in mice that received vehicle, and this was reduced to ~5% in mice that received uPSEM792s (Fig. 6D). These results indicate that approximately half of the fear recall ensemble cells were reactivated during subsequent recall of the memory 2 weeks later, and uPSEM792s was highly effective in blocking this reactivation.

## Discussion

Pharmacological, chemogenetic, and optogenetic experiments have demonstrated that the dmPFC is a key site for driving cocaine seeking [31, 43–45]. Previous studies have demonstrated that inactivation of a small ensemble of neurons (<10% of total) in the dmPFC is sufficient to disrupt future cocaine seeking [46, 47]. Here, we provide further evidence on the stability, necessity, and specificity of a dmPFC cocaine seeking ensemble. Using two different tagging strategies we found that ~40% of the original neuronal ensembles were reactivated in the dmPFC in a subsequent cocaine seeking session 2 weeks later. The percentage of the tagged ensemble that was reactivated during the second drug seeking test positively correlated with the persistence ratio of cocaine seeking in the dmPFC, underscoring the significance of the dmPFC in maintaining long-term cocaine seeking memory. These results prompted us to test the necessity and specificity of the dmPFC cocaine seeking ensemble. In addition to its importance in cocaine seeking, the dmPFC is also a crucial regulatory hub for fear memory retrieval [15, 47–51]. In the current study, we used inhibitory chemogenetic tools to selectively inactivate dmPFC neuronal ensembles that were previously activated during either cocaine seeking or cued recall of a fear memory. Inhibition of a dmPFC cocaine seeking ensemble suppressed cocaine seeking 2 weeks later but did not affect locomotor activity or fear memory retrieval. This specificity was also observed with the dmPFC fear ensemble. Inhibition of the dmPFC fear recall ensemble reduced the expression of conditioned fear 2 weeks later but did not affect locomotion or cocaine seeking. Additionally, the dmPFC ensembles encoding cocaine-seeking and the cued fear memory are similar in size but exhibit significantly low overlap. Taken together, this indicates that the ensembles encoding different types of memory recall are composed of distinct dmPFC neurons.

Several subregions of the prefrontal cortex have been identified as containing drug seeking ensembles [52]. In an early study, Bossert et al. inactivated a vmPFC ensemble engaged during exposure to a heroin seeking ensemble and found that context-induced reinstatement of heroin seeking was reduced [50]. A heroin seeking ensemble was also identified in the orbitofrontal cortex, where inactivation of this ensemble after incubation of craving suppressed drug seeking [51]. There is also evidence that the prefrontal cortex forms ensembles that can suppress drug seeking. Inactivation of a vmPFC ensemble recruited by exposure to alcohol cues led to greater levels of intake [53]. Further support comes from the finding that separate ensembles for cocaine seeking and extinction have been identified in the vmPFC [54, 55]. In addition to forming specific ensembles for different behaviors associated with the same reinforcer, the prefrontal cortex can form generalized reward seeking ensembles. Pfarr et al. found that operant sucrose and alcohol seeking engaged ensembles with ~50% overlap within the vmPFC [52], suggesting that a common ensemble may promote seeking for both rewards. Using the same reinforcer trained in distinct contexts, Jessen et al. identified an ensemble within the dmPFC whose reactivation was positively associated with persistence of goal-directed sucrose seeking across the two contexts [32].

The prefrontal cortex is also a site where ensembles encoding a fear memory have been identified. The Luo lab demonstrated that a dmPFC fear retrieval ensemble is required for fear memory retrieval 28 days following conditioning when the ensemble was tagged during a retrieval test 7 or 14 days, but not 1 day after conditioning, suggesting that the ensemble involved in recent recall is not recruited by remote memory, but a remote ensemble remains stable [29]. In contrast, we found that inhibition of the dmPFC fear recall ensemble tagged only 2 days following conditioning reduced recall 2 weeks later, suggesting that this ensemble forms shortly after 1 day. An additional study found that inhibition of a dmPFC ensemble tagged during fear conditioning reduced fear conditioned suppression of food seeking 28 days later, however this effect was observed in females, but not males [56]. Teng et al. found that activity within the dmPFC ensemble was also required for updating the original memory. Optogenetic inhibition of this ensemble during a recall test reduced recall, but spontaneous recovery after extinction was increased, indicating that the dmPFC ensemble tagged during recall was required for memory updating [57]. Taken together, our data support earlier studies demonstrating that dmPFC ensembles are required for remote recall of fear-related memories.

There are some limitations to the current work. First, the timelines of the experiments tagging cocaine seeking and fear conditioning were different, such that fear conditioning was performed before the final cocaine seeking test in the fear tagging experiment. Fear and fear-induced stress can influence the motivation to seek drugs like cocaine [58, 59]. This temporal distinction may underlie the greater variability observed in day 21 drug seeking in the fear tagged mice. However, we saw no differences in day 21 cocaine seeking after inhibition of the fear recall ensemble. Although both sexes of mice were used, experiments were not adequately powered to detect sex differences. Outcome measures were similar between sexes within each group, and in cases where ensemble inhibition reduced a behavior, this effect was observed in both sexes. Additionally, the use of a 2 h seeking session could have contributed to tagging both cocaine seeking- and extinction-promoting cells. In the current and prior studies, we have found that mice show little within-session extinction of responding during 2 h reward seeking sessions (Supplementary Fig. 1A) [32]. Furthermore, inhibition of a prefrontal cocaine extinction ensemble has been previously shown to increase seeking [54], which is contrary to the effect reported here. Nonetheless, we cannot exclude the possibility that our tagging strategy included cells that encode extinction.

Considering the results, it is difficult to determine the precise nature of what the dmPFC cocaine seeking ensemble cells encode. For example, is the “desire” to obtain cocaine suppressed, or does ensemble inhibition simply suppress the learned motor program previously required to obtain the drug? Evidence against the latter possibility comes from data showing that non-selective inhibition of dmPFC does not interfere with operant responding during a visual cue discrimination task [60], indicating that dmPFC activity is not required to execute an operant motor program. It is also unknown whether the dmPFC cocaine seeking or fear recall ensemble cells interact with cells that encode extinction of the learned behavior. Stated another way, does inhibition of the dmPFC ensemble prevent cocaine seeking/fear recall by promoting extinction? This seems more likely for cocaine seeking, since most mice exhibited hundreds of operant responses under extinction conditions prior to the test day. However, ablation of a dmPFC ensemble that was tagged with a brief exposure to a cocaine seeking environment (which did not lead to extinction of seeking in subsequent testing) was sufficient to suppress drug seeking [28], suggesting that significant extinction learning is not required for inhibition of the dmPFC ensemble to suppress drug seeking.

To conclude, we showed the necessity and specificity of both a dmPFC cocaine seeking and a conditioned fear ensemble in remote recall of each behavior. Our investigation revealed that the cocaine seeking ensembles and cued-fear ensembles recruited comparable cell proportions (~8%) within the dmPFC, and these two ensembles are largely non-overlapping. Differences at the cellular level are also directly reflected in behavior. Inhibition of the cocaine seeking ensemble suppressed cocaine seeking without impacting recall of fear memory. Conversely, inhibiting the cued fear ensemble reduced conditioned freezing while not influencing cocaine seeking. The persistent nature of drug seeking and recall of fearful memories represents a significant obstacle for the treatment of substance use disorders and post-traumatic stress disorder. Our results suggest that strategies to target dmPFC ensembles associated with recall of drug or fear-related memories could have effectiveness without having a broad impact on memory recall or dmPFC function.

## Supplementary information

Supplemental figure captions

Supplementary methods

Supplementary Figure 1

Supplementary Figure 2

## Data Availability

The data underlying this article will be shared on reasonable request to the corresponding author.
