# Supplementary material for: Dissociable dorsal medial prefrontal cortex ensembles are necessary for cocaine seeking and fear conditioning in mice
Source: Transl Psychiatry. 2024 Sep 23;14:387. doi: 10.1038/s41398-024-03068-7 (PMC11420216; doi:10.1038/s41398-024-03068-7)
Supplement: Supplementary file 1 — Supplemental figure captions [file 41398_2024_3068_MOESM1_ESM.docx]

**Supplemental figure 1: Cumulative lever responses during the early (day 7 seeking) and late cocaine seeking (day 21 seeking) sessions.** The cumulative lever responses during each seeking session revealed that seeking endured through the duration of each session, validating our choice to tag the full 2-hour sessions.

**Supplemental figure 2: Active and inactive lever responding over the first seven and last three SA sessions in fear recall tagged mice.** Active lever presses are denoted as solid symbols, inactive lever presses are denoted as hollow symbols. Linear mixed effects analysis found no effect of tag, ligand, or interaction (all p>0.94).
