# Supplementary material for: Dissociable dorsal medial prefrontal cortex ensembles are necessary for cocaine seeking and fear conditioning in mice
Source: Transl Psychiatry. 2024 Sep 23;14:387. doi: 10.1038/s41398-024-03068-7 (PMC11420216; doi:10.1038/s41398-024-03068-7)
Supplement: Supplementary file 2 — Supplementary methods [file 41398_2024_3068_MOESM2_ESM.docx]

**Supplementary Methods**

**Subjects:**

Male and female TetTag histone H2B-EGFP mice [10-24 weeks; experimental: n = 14 (6-8/sex)] and *c-fos*-tTA mice [8-16 weeks old; n = 66 (8-9/group)] were generated in house for use in this study. The TetTag double transgenic line was generated by crossing a hemizygous transgenic mouse that express a histone H2B-EGFP fusion protein controlled by the tetO promoter (Jackson Laboratory strain #005104) with mice that express tetracycline-transactivator (tTA) protein under control of the *c-fos* promoter (Jackson Laboratory strain #018306). The cfos-tTA mice were obtained from the Jackson Laboratory (Strain #018306). Males expressing both the c-fos-tTA and the tetO-H2B/EGFP transgenes were paired with female C57BL/6J mice to generate the cfos-tTA mice and double positive TetTag mice used in this study. Mice were born and raised on doxycycline (dox) chow (40mg/kg, Teklad Custom Research Diets, cat#120240, Envigo, Harlan Laboratories, Madison, WI) to prevent EGFP expression. After weaning, Mice were maintained, and group housed on a 12-h reverse light cycle with food and water available *ad libitum*. Subsequently, mice were transferred to single housing and underwent handling for three consecutive days prior to the commencement of experiments. The TetTag mouse line and c-fos-tTA mouse line employed in this study are both Tet-off systems. In the absence of dox, activation of the c-fos promoter triggers the expression and “tagging” of a stable H2B-EGFP fusion protein that persists for multiple weeks after induction. Thus, tagging is accomplished by removal of dox from the diet and resuming dox to stop tagging. Conversely, the presence of dox prevents the expression H2B-EGFP. For TetTag mice, dox chow was removed and replaced with regular chow for 3 days prior to the tagging session to open the window for activity-dependent labeling. However, based on our pilot results, a robust tagging response in cfos-tTA mice could be achieved by removing dox only one day prior to the tagging session. Therefore, the timing of dox removal was adjusted accordingly for *c-fos*-tTA mice, differing from the approach used for TetTag mice. All experimental protocols were approved and conducted in compliance with the Medical College of Wisconsin (MCW) Institutional Animal Care and Use Committee in accordance with the NIH guidelines for the care and use of laboratory animals.

**Viral constructs and stereotactic surgery**

To label the initial phase of cocaine seeking ensemble in the dmPFC of c-fos-tTA mice, a co-injection of AAV_5_-hsyn-FLEX-PSAM-GlyR-IRES-EGFP (Addgene, #119741) and AAV1-TRE-Cre (SignaGen Laboratories, #SL101511) was conducted before jugular surgery.

Mice undergoing stereotactic injections were anesthetized under isoflurane according to established protocols (5% induction, 1.5-3% maintenance). Standard stereotactic procedures were used. For injections into the dmPFC, the coordinates used were as follows: AP 1.7 mm; ML ± 0.4 mm; DV 2.2 mm. AAVs were injected in a volume of 400 nl using a mineral oil-filled glass micropipette attached to a Nanoject III programmable Nanoliter Injector (Drummond Scientific Company, Broomall, PA). The injection rate was set at 60 nl/min, and the injectors were left in place for 5 min to ensure adequate diffusion from the injection tip. After completion of the surgical procedure (before recovery of consciousness), mice were administered Rimadyl (Carprofen, 5 mg/kg) through subcutaneous injections as an analgesic. Mice then were placed on a heating pad until full recovery from anesthesia. A minimum recovery period of 7 days was given prior to Jugular catheterization surgery. The histology of all injections was verified after behavior experiments.

**Jugular catheterization surgery**

Approximately 7 days after stereotaxic surgery, mice were catheterized. Briefly, mice were anesthetized with a cocktail injection of ketamine HCl (10 mg/kg, i.p) and xylazine (0.2 mg/kg, i.p) and were implanted with a silicone catheter into the right jugular vein, which exited through the intrascapular region and was connected to a cannula assembly. The cannula assembly consisted of a stainless-steel cannula (Plastics One, Roanoke, VA) mounted on a silicone base with nylon mesh and dental acrylic, which was implanted subcutaneously. Rimadyl (Carprofen, 5 mg/kg) was administered immediately following completion of the surgical procedure (before recovery of consciousness) and 24-h later. Catheters were flushed with 0.1 ml of heparinized saline (100 unit/ml, Human Pharmaceuticals), both 3 days after surgery and 1 day before self-administration. Mice were allowed to recover ≥ 7 days prior to the start of self-administration experiments.

**Drugs**

Cocaine-HCl (NIDA Drug supply program) was dissolved in 0.9% saline (Midwest Vet Supply, Lakeville, MN). A stock solution of cocaine at 2 mg/ml was prepared for jugular infusions. This stock solution was freshly prepared on the first day of cocaine self-administration. The remaining solution was stored at 4 ℃ blocked from light. Any unused drug was discarded after 2 weeks. uPSEM792s hydrochloride (Hello Bio, #HB8542) ligand was dissolved in sterile saline and prepared on the day of use at a dosage of 0.3 mg/kg via intraperitoneal injection.

**Cocaine self-administration**

Self-administration experiments were performed within in a sound-attenuating box, using an operant conditioning chamber (21.6 x 17.8 x 12.7 cm, Med Associates). All chambers were equipped with two retractable levers on each side of the left wall. Additionally, a yellow LED light was mounted above each lever.

After the mice had fully recovered from the jugular catheterization surgery, they were processed to undergo cocaine self-administration on a fixed ratio-1 (FR-1) for 7-14 days. All self-administration chambers and levers were wiped with a 30% ethanol solution prior to the start of each session. Each self-administration session commenced with the starting of the fan, extension of the two levers, and a single noncontingent dose of cocaine (0.5 mg/kg/infusion) with presentation of the cue light above the active lever (5 seconds). During the self-administration session, a single press on the active lever resulted in the delivery of cocaine and cue light illumination, followed by a 10 second time out period during which active lever presses did not result in drug or cue delivery. Presses on the inactive lever were recorded but had no programmed consequence. If no active lever presses occurred within the initial 30 minutes of the session, an additional noncontingent dose of cocaine was administered. The self-administration sessions were limited to a maximum of 64 reinforcers and ran for a total duration of three hours, unless the mice reached 64 infusions prior to the end of the session. Cocaine self-administration training continued until specific criteria were met (four consecutive sessions of > 20 reinforcers and a 2:1 ratio of active to inactive lever presses, minimum of seven sessions). If criteria were not met following the initial seven sessions, catheter patency was assessed using Brevital (9mg/kg, iv) and any mouse not meeting criteria for patency (sedation within 5 seconds) was excluded from the study. Self-administration sessions were conducted for a maximum of 14 days.

**Cocaine seeking**

Once cocaine self-administration criteria were met, all mice underwent 6 days of forced abstinence where they remained in their home cage with no access to the self-administration chambers or cocaine. In the case of TetTag mice, on the fourth day of abstinence, all mice were removed from the dox diet and placed on a regular chow until after the early drug seeking session. For the *c-fos*-tTA mice, dox chow was replaced by regular chow on the sixth day of abstinence for mice from tagged groups; for mice from non-tagged groups, mice remained on the dox throughout the study. On the seventh day, mice were placed back in the operant chambers and underwent a two-hour drug seeking session under extinction conditions, during which the ensemble was tagged. This seeking session was under the same conditions as cocaine self-administration (pump and cue light), except there was no cocaine infusions were delivered and sessions were two hours in duration, regardless of lever presses. Following the seeking session, mice were returned to home cages and provided immediate access to the dox diet to close the tagging window. The mice then underwent another 13 days of forced abstinence. On the 21^st^ day of abstinence, all mice underwent a second cocaine seeking session. This session was identical to the one performed on day 7. For cfos-tTA mice, the uPSEM792s ligand (0.3 mg/kg) or vehicle was administered 30 min prior to the start of the second seeking session.

**Slice preparation and electrophysiology**

Twenty-four hours after the day 21 seeking session, two of the cfos-tTA mice were sacrificed and brain slices containing the medial prefrontal cortex were prepared. Mice were anesthetized with isoflurane inhalation and decapitated. The brain was then embedded in low-melting-point agarose, and 200 µm thick coronal slices were prepared using a vibrating microtome (Leica VT1200s). The slicing process was performed in a N-Methyl-D-glutamine (NMDG) -based solution containing the followings (in mM): 92 NMDG, 26 NaHCO_3_, 2.5 KCl, 1.25 NaH_2_PO_4_, 0.5 CaCl_2_-2H_2_O, 7 MgSO_4_-7H_2_O, 20 HEPES, 25 Glucose, 2 Thiourea, 5 Na-ascorbate, and 3 Na-pyruvate. Following the slicing, artificial cerebrospinal fluid (ACSF) was gradually introduced into the NMDG solution at room temperature, with incremental additions occurring every 5 min over a span of 20 min. The ACSF solution contained the following (in mM): 119 NaCl, 3 KCl, 2 CaCl_2_-2H_2_O, 1.3 MgCl_2_-6H_2_O, 1.25 NaHPO_4_, 25 NaHCO_3_, and 10 Glucose. The slices were allowed to recover for at least an additional 30 min in ACSF prior to recording. All solutions were oxygenated with 95% O_2_ and 5% CO_2_. Neurons were voltage-clamped at -70 mV and then current-clamped once the membrane potential became stable. Glass pipettes filled with an internal solution containing the following (in mM) were used for the recordings: 140 K-gluconate, 10 KCl, 10 HEPES, 0.2 EGTA, 2 MgCl_2_, 4 Mg-ATP, 0.3 NA_2_-GTP, 10 Na_2_phoshpocreatine. A 20-pA current injection was applied to induce stable action potential firing in the neurons. Pressure injection of uSPEM792s ligand (50 nM) was given via a glass pipette (1-2 µm tip opening, 5 psi, 2 s). All recordings were conducted at 31 ± 1 ℃ using an automatic temperature controller (Warner Instruments, Inc.).

Whole-cell recordings were conducted from dmPFC neurons with strong EGFP fluorescence or without EGFP fluorescence as visualized under infrared differential interference contrast video microscopy with epifluorescence using a patch-clamp amplifier (Multiclamp 700B). Data acquisition and analysis were performed using DigiData 1440A and 1550B digitizers, along with pClamp 10 (Molecular Devices) analysis software. The signals were filtered at 2 kHz and sampled at 10 kHz.

**Open field test**

To assess the potential impact of temporal inhibition of dmPFC cocaine ensemble activity on locomotor activity and sedation during the second drug seeking session in cfos-tTA mice, an open field test was conducted 24 hours after the day 21 cocaine seeking session. Mice were placed individually into the center of a round plexiglass arena (diameter 47 cm, height 33 cm) and allowed to freely explore the arena during a 2-h test session. Locomotor activity was tracked and recorded using a Sony Handycam camera (HDR-CX405) mounted in the ceiling above the arena to monitor position and movement of the animals. Total distance travelled, entries into center and immobile time were calculated through an automated video-tracking system (ANY-maze, Stoelting, Wood Dale, IL). The center was defined as the central 23.5 cm diameter area of the open field chamber. 30 min prior to the assay, the mice received either an uPSEM792s ligand (0.3 mg/kg) or a vehicle.

**Fear conditioning**

Three days after the open field assay, mice were placed individually into the fear conditioning chamber (30 cm x 30 cm x 25 cm, Actimetrics, Wilmette, IL) within a sound- and light- attenuating box. The chamber was illuminated by a 25 W yellow bulb for the training. The experimental contingencies were controlled by a computer via FreezeFrame software (Coulbourn). Initially, mice were placed in the chamber for 3 min before presenting a 20 s, 80 dB, and 420 Hz tone. During the last 2 s of the tone, a 0.5 mA footshock was delivered via the grid floor. The pairing was repeated 7 times with a 200 s interval between each repetition to reinforce the association between the tone and the shock. On the following day, mice were tested under the same context as the training session without foot shocks for 6 min to assess the contextual memory. Prior to the test. mice from different groups received the uPSEM792s ligand or vehicle 30 min in advance. Following a 24 h interval from the contextual test, mice were assessed in a novel context. The fear conditioning chambers’ walls and floor were coved with black plastic surfaces sprayed with 1% acetic acid, and the illuminating light was switched to red. During the cued test, mice were exposed to 7 tones, each lasting 20 s, with a 200 s interval between each presentation. No foot shocks were administered. Like previous sessions, uPSEM792s ligand or vehicle were given to animals 30 min prior to the test. In all sessions, the percentage of freezing time during the assay or tone presentations was measured.

**Immunohistochemistry and image analysis**

Immediately following the second cocaine seeking session or 1-hr after the cued fear test, mice were subjected to transcardial perfusion with 0.01 M PBS followed by 4% paraformaldehyde (PFA). Subsequently, brains were post-fixed in 4% PFA overnight and then stored in 30% sucrose/PBS solution until sectioning. Coronal sections (20 μm) were obtained using a cryostat. The free-floating sections were first washed in 0.01 M PBS and then blocked with a solution of 1% BSA/0.4% Triton X-100/0.01 M PBS for 1 hour. Next, the sections were incubated for 48 hours at 4°C in a solution containing antibodies for c-Fos (1:2000, Synaptic Systems, #226-003). This c-Fos antibody is specific and does not recognize other fos-related antigens such as delta-FosB. After rinsing with 0.01 M PBS, the sections were incubated with a donkey anti-rabbit Cy3 secondary antibody (1:1000, Jackson Immuno, #711-166-152) for 90 minutes at room temperature. Following another wash with 0.01 M PBS, the slices were incubated with 0.002% DAPI in 0.01M PBS for 30 minutes. Finally, sections were rinsed with 0.01 M PBS, mounted on slides, and coverslipped with Vectashield mounting medium (Vector Laboratories).

Prefrontal cortex sections in all the experiments were imaged using a Leica SP8 confocal microscope at 1024 x 1024 resolution under a HC Fluro L 25x/0.95 water immersion objective with identical parameters. Images on both sides of sections were captured. Four sections per animal were imaged. For each brain section, images were cropped to capture regions of prelimbic and infralimbic of cortex. Images were analyzed automatically using Imaris 10.0 software (Bitplane/Oxford Instruments) via custom Imaris algorithms to count the EGFP+ and c-Fos+ nuclei with the spot function. The percentage of c-Fos+ neurons (c-Fos+/DAPI%), EGFP+ neurons (EGFP+/DAPI%), and percentage of EGFP reactivation, quantifies overlapping ensemble presence with respect to the EGFP+ population (EGFP+*c-Fos+/EGFP+%), and cell counts of co-localized spots between EGFP+ and c-Fos+ were quantified. Co-localization of spots was performed using a threshold of 4 µm.

**Statistical analysis**

Prism10 (GraphPad, San Diego, CA, USA) or SPSS 28 (IBM, Armonk, NY) were used for statistical analyses. Data were analyzed by Student’s t-test, linear regression, ANOVA (repeated measures when appropriate), linear mixed effect analysis, or ANCOVA followed by Holm-Sidak multiple comparisons tests. All data are expressed as mean ± SEM. In all tests, a value of p < 0.05 was considered significant.
