# Supplementary figures and images for: Dissociable dorsal medial prefrontal cortex ensembles are necessary for cocaine seeking and fear conditioning in mice
Source: Transl Psychiatry. 2024 Sep 23;14:387. doi: 10.1038/s41398-024-03068-7 (PMC11420216; doi:10.1038/s41398-024-03068-7)

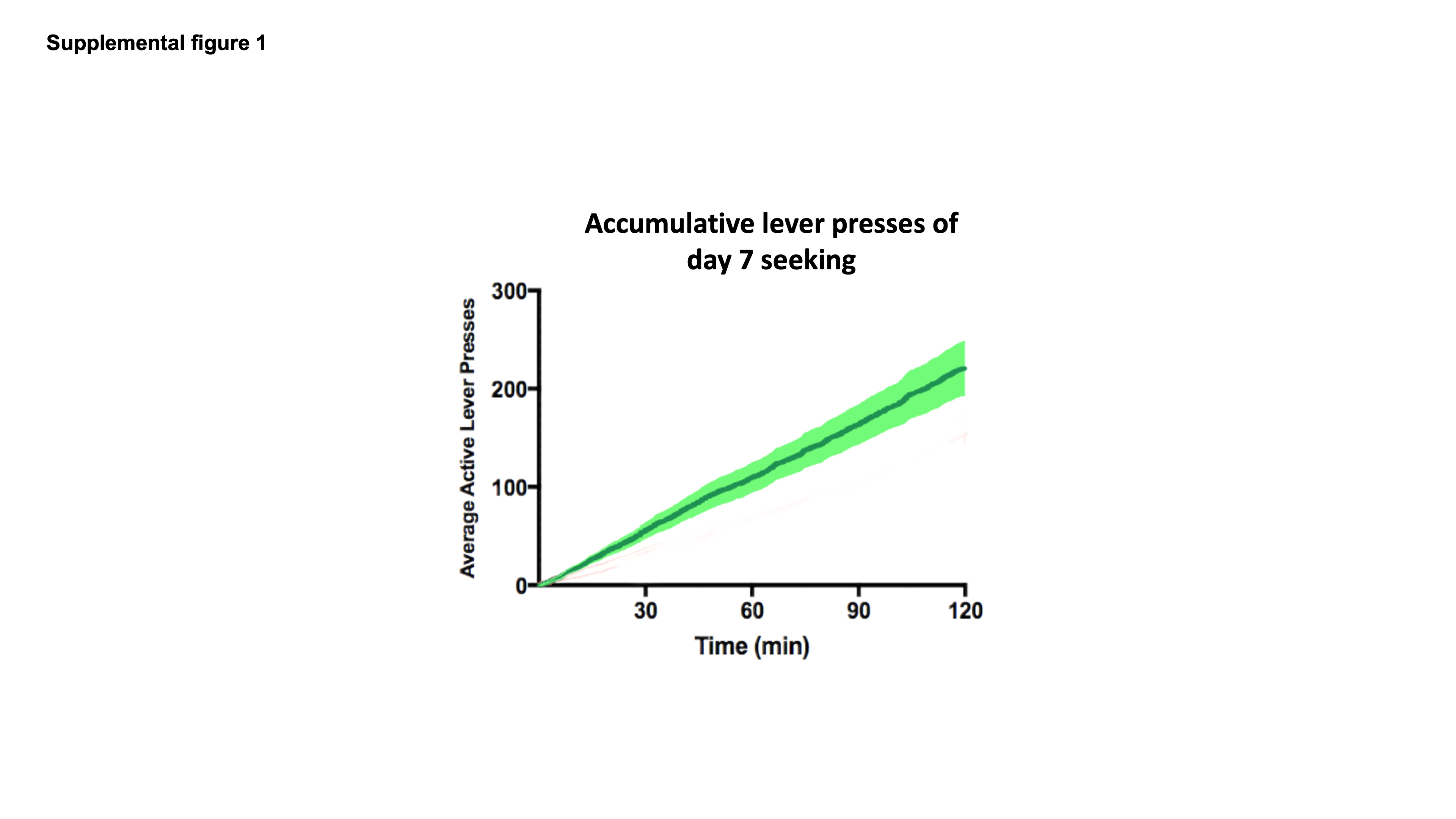

Supplement: Supplementary file 3 — Supplementary Figure 1 [file 41398_2024_3068_MOESM3_ESM.tif]

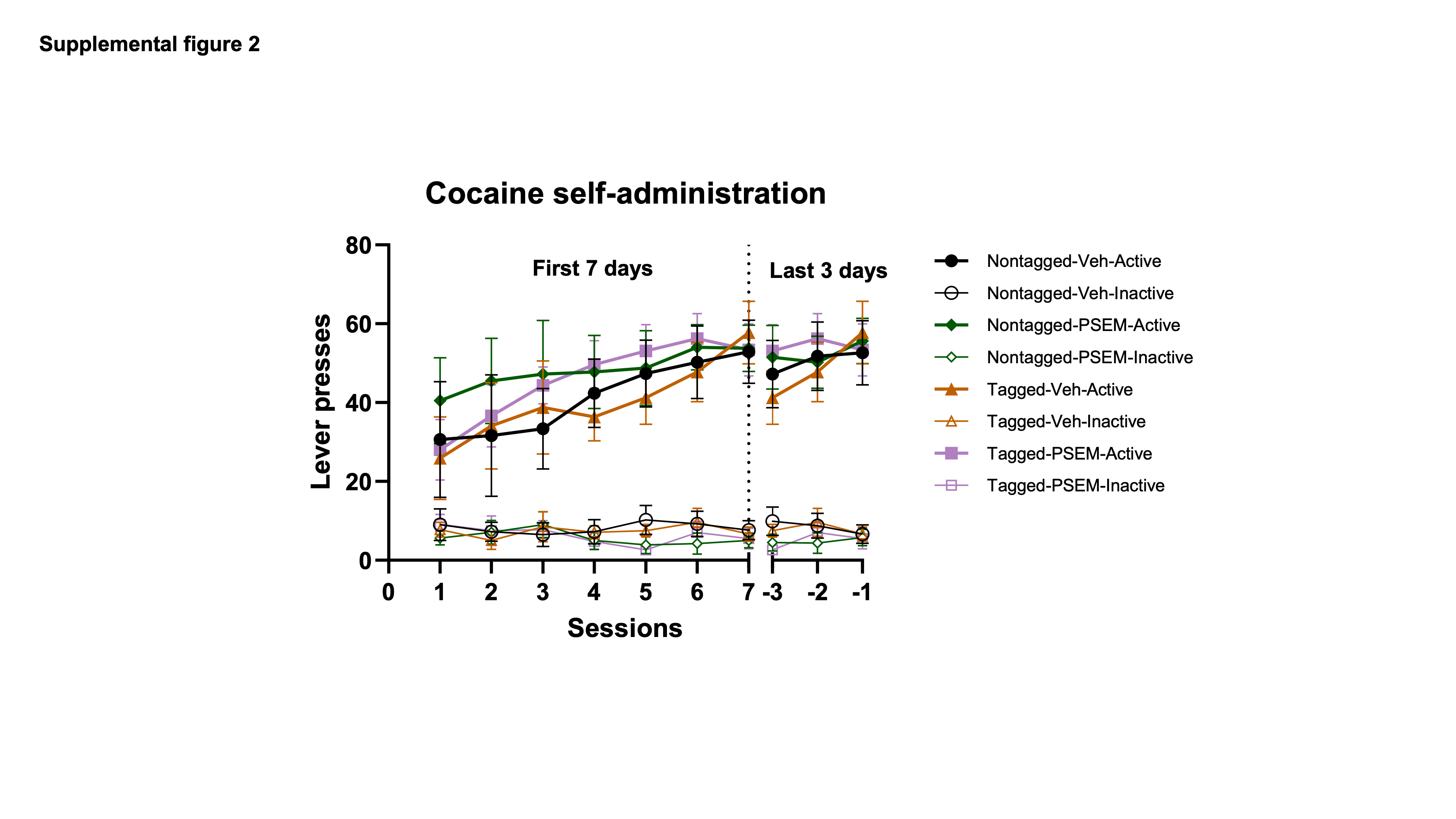

Supplement: Supplementary file 4 — Supplementary Figure 2 [file 41398_2024_3068_MOESM4_ESM.tif]
